# Sweet fruit from a poisonous kiss

**DOI:** 10.1186/1756-8722-3-19

**Published:** 2010-05-28

**Authors:** Delong Liu

**Affiliations:** 1 MD, PhD, Professor of Medicine, New York Medical College, Valhalla, NY 10595, USA

## Editorial

Acute promyelocytic leukemia (APL), once a rapidly fatal disease, has become most curable of all leukemias, thanks to the two Chinese wonder drugs, all-trans retinoid acid and arsenic trioxide (AT). PML-RXARα fusion protein is the hallmark and driving force of APL. In the April 9^th ^issue of the journal, *Science*, Zhang *et al*. provided new and convincing evidence on how AT, an ancient poisonous medicine, plays the tricks on the oncoprotein, PML-RARα, and sheds lights on how a poisonous kiss leads to the sweet fruit [1].

## A comprehensive approach and an epic story of science

Zhang *et al*. have indeed done a tremendous amount of work in this study. Several cell lines and transfectants were used and/or established, including HEK 293T, NB4, and HeLa cells, to name a few. Numerous mutants of PML as well as EGFP-fusion proteins were generated to pinpoint the kissing spot of AT. To be even more convincing, two additional organic arsenicals, PAPAO, and ReAsH, were also employed. With collaborations from the leading institutions of biotechnology and physics in China, MALDI-TOF mass spectrometry, EXAFS, XANES spectroscopy, and circular dichroism were orchestrated marvelously into the study. As the study evolves, it reads like an epic story of science.

## Solid evidences-with no stones left unturned

The scientific team has provided strong and convincing evidence step by step and proved to the world that AT poisons the PML-RARa protein by kissing the cysteine residues in the zinc fingers within the RBCC domain of PML. They first demonstrated that AT binds to PML-, not the RARα - portion of the fusion oncoprotein. Using EGFP-fusion mutants of PML protein, they showed colocalization of arsenicals with various PML mutants, and pinpointed that AT binds to the RING domain of PML. With the help of NMR and a variety of technologies of physics, arsenic-sulfur bonds were proven to form through the conserved cysteine residues, and the number of arsenic atoms bound to the PML peptides were also defined. The biological consequences of arsenic binding to PML, the enhanced interaction of PML with UBC9 leading to increased degradation of PML-RARα, were further explored both in vitro and in vivo. In the end, a working model was proposed to explain how the poisonous kiss controls the fate of PML-RARα and leads to the sweet cure of APL.

## Masterful collaborations between leading Chinese institutions and international laboratories

As soon as you read the study, it is obvious that the success of this remarkable work proves again the importance of collaborations between major leading institutions in China as well as with international laboratories. With the vast experience and knowledge from decades long of scientific endeavors in the world-renowned laboratories of Drs. Zhu Chen and Sai-Juan Chen, the collaborations between leading Chinese institutions and international laboratories were skillfully mastered to utilize the necessary tools and technology and generate the convincing and authoritative scientific data. This sets up a historical example in China for collaborations among leading laboratories and institutions to embark on scientific frontiers and achieve international leadership quickly and effectively.

## References

[B1] Zhang X-W Yan Xiao-Jing Zhou Zi-Ren Yang Fei-Fei Wu Zi-Yu Sun Hong-Bin Liang Wen-Xue Song Ai-Xin Lallemand-Breitenbach Valérie Jeanne Marion Zhang Qun-Ye Yang Huai-Yu Huang Qiu-Hua Zhou Guang-Biao Tong Jian-Hua Zhang Yan Wu Ji-Hui Hu Hong-Yu de Thé Hugues Chen Sai-Juan Chen Zhu Arsenic trioxide controls the fate of the PML-RARα oncoprotein by directly binding PML Science 2010 328 240 243 10.1126/science.1183424 20378816

